# Reading-Related Skills Associated With Acquisition of Chinese as a Second/Foreign Language: A Meta-Analysis

**DOI:** 10.3389/fpsyg.2022.783964

**Published:** 2022-03-16

**Authors:** Xi Chen, Jingjing Zhao

**Affiliations:** ^1^Faculty of Education, Shaanxi Normal University, Xi’an, China; ^2^Mental Health Education Center, Chengdu University, Chengdu, China; ^3^School of Psychology, Shaanxi Normal University, Xi’an, China

**Keywords:** Chinese as a second/foreign language, Chinese word reading, meta-analysis, CSL/CFL learners, reading-related skills

## Abstract

Previous studies have found the effect of cognitive skills (e.g., phonological awareness, morphological awareness, orthographic awareness, and rapid automatized naming) on reading ability, but the role of different reading-related skills in acquisition of Chinese as a second/foreign language (CSL/CFL) remains unexplored. Prior meta-analyses on the relationship between cognitive skills and reading have been conducted primarily in native English-speaking or Mandarin-speaking children. The purpose of the present meta-analysis was to examine the relationship between Chinese reading-related skills and Chinese word reading of CSL/CFL learners. A search of English and Chinese databases yielded 42 effect sizes, comprising 1103 subjects met the criteria for meta-analysis and were included in the final meta-analysis. Results revealed a moderate relationship between phonological awareness (*r* = 0.41), morphological awareness (*r* = 0.36), orthographic awareness (*r* = 0.38), rapid automatized naming (*r* = −0.32) and Chinese word reading in CSL/CFL learners. In addition, a moderating effect of length of study on the relationship between phonological awareness and Chinese word reading (*Q_*B*_* = 5.20, *p* = 0.023): phonological awareness and Chinese word reading correlated more strongly for beginning learners than for advanced learners. These results suggest importance of cognitive factors in the acquisition of Chinese word reading as a second language. Results also shed light on the impact of length of study on the influence from phonological awareness to the sensitive period of phonological learning for CSL/CFL learners.

## Introduction

Chinese has become more and more important as a second or foreign language (CSL/CFL) both inside and outside China over the last two decades. According to the official website of the Central People’s Government of the People’s Republic of China, more than 4,000 universities, 30,000 primary and secondary schools, and 45,000 Chinese language schools and training institutions worldwide offer Chinese language courses, and the cumulative number of people learning Chinese outside of China had reached 200 million by September 2020^1^. The expansion of CSL/CFL teaching and learning has been linked to extensive study on topics involving the learning of Chinese in non-Chinese learners across the world (Gong et al., 2020). Learning to read Chinese, a logographic writing system (Shu, 2003), is a unique process as compared to learning to read an alphabetic language such as English.

Learning to read is a process that requires a number of cognitive and metalinguistic skills (Tong and McBride-Chang, 2010a). Previous studies have shown that phonological awareness (PA), morphological awareness (MA), orthographic awareness (OA), and rapid automatized naming (RAN) were particularly essential for children’s reading acquisition, both in alphabetic languages and in Chinese reading (McBride-Chang and Manis, 1996; Ho and Bryant, 1997a; Parrila et al., 2004; Tong et al., 2009; Tong and McBride-Chang, 2010b; Lei et al., 2011; Li et al., 2012; Lin et al., 2012; Xue et al., 2013; Wang et al., 2014). But their roles are slightly different in Chinese reading and English reading. For example, it has been argued that PA plays a more important role in English reading, while MA plays a distinct role in Chinese reading (McBride-Chang et al., 2006, 2013; Shu et al., 2006). The influence of these cognitive skills on reading in native languages (e.g., English and Chinese) have been systematically investigated in previous meta-analyses (Swanson et al., 2003; Melby-Lervåg, 2012; Song et al., 2016; Ruan et al., 2018). However, no systematic review or meta-analysis has been done to examine the relationship between these cognitive skills and Chinese reading in CSL/CFL learners. The purpose of this meta-analysis was to investigate the relationship between reading-related skills and reading of Chinese as a second/foreign language.

### Characteristics of Chinese Characters

More than 80 percent of modern Chinese characters are phonetic-semantic compounds [e.g., 材/cai2/(material)], i.e., a character is made up of two parts: a phonetic radical and a semantic radical. The phonetic radical provides the pronunciation clues (e.g., 才/cai2/) and the semantic radical provides the meaning information [e.g., 木(wood)] (Shu, 2003). In terms of the semantic radical, according to Shu et al. (2003)’s research, about 58% of the characters taught in elementary school are semantic transparent [the semantic radical represents the character’s conceptual category, e.g., 吃(eat), the semantic radical 口(mouth) is directly related to the meaning of the character], 30% are semi-transparent [the semantic radical indirectly relates to the character’s meaning, e.g., 猎 (hunting), the semantic radical 犭 means animal] and 9% are opaque characters [the radical gives no clues regarding the character’s meaning, e.g., 略 (brief), the semantic radical 田(field) provides no information about character meaning]. For the phonetic radical, about 39% of characters are regular (the phonetic radical provides trustworthy information about the pronunciation of a character, e.g., 粮/liang2/, the phonetic radical is 良/liang2/), about 26% are semi-regular (the phonetic radical provides only limited information about the pronunciation, e.g., 积/ji1/ with the phonetic radical 只/zhi1/) and 15% are irregular characters (the phonetic radical provides no clues regarding the pronunciation, e.g., 路/lu4/ with the phonetic radical 各/ge4/). In addition, McBride (2016) also argues that Chinese character learning differs in four fundamental ways (semantic radicals, morphology and phonology, scripts, and orthography) from alphabetic language learning. Chinese as a logographic language, does not have the same grapheme–phoneme correspondence (GPC) rules as alphabetic languages (McBride, 2016). Furthermore, in Chinese, one character typically represents one morpheme, and there are a large number of homophones [e.g., 意/yi4/(meaning) and 异/yi4/(different)]. Thus learning Chinese is more challenging for L2 Chinese learners than L2 alphabetic language learners, especially for native speakers of alphabetic languages.

### Cognitive Skills in Chinese Character Reading

It is well-accepted that PA and MA were both important for Chinese reading acquisition. PA is one category of metalinguistic development that continues to get attention as an important component of early reading abilities (Ball and Blachman, 1988). It is the ability to identify that a spoken word consists of a series of sounds, as well as to reflect on and manipulate the spoken language’s subunits, phonemes and words (Tunmer et al., 1988). MA implies an understanding of morphemes, either implicitly or explicitly (McBride-Chang et al., 2003), and a morpheme is the smallest unit of meaning and grammatical function in a language.

In terms of PA and MA’s contribution to Chinese acquisition, previous studies have consistently found they were important in Chinese reading (Ho and Bryant, 1997b; Siok and Fletcher, 2001; Li et al., 2002; McBride-Chang et al., 2003, 2005; Zhou et al., 2012; Hu, 2013; Tong et al., 2017). However, some studies found both PA and MA were important in Chinese L1 reading (Li et al., 2002; Hu, 2013), while others stressed the higher effect of MA than PA in both Chinese L1 (McBride-Chang et al., 2005; Zhou et al., 2012; Tong et al., 2017) and Chinese L2 reading (Hao and Zhang, 2006). It should be noted that in terms of the role of PA for Chinese reading, Shu et al. (2008) highlighted the significance of tone and syllable awareness in Chinese children’s early character acquisition, whereas Siok and Fletcher (2001) found onset-rime awareness played an important role in Chinese reading.

Previous research has also revealed OA plays an essential role in Chinese acquisition (Ho et al., 2003; Tong et al., 2009; Georgiou et al., 2021). OA refers to the understanding of the print conventions employed in a writing system or knowledge of how words are spelled (Conrad et al., 2013). Shaped like squares, Chinese characters which are made up of strokes emphasize visual structure and arrangement (Ho et al., 2003; Tong and McBride-Chang, 2010a). Therefore, Chinese OA is distinctly different from alphabetic languages, which is the ability to identify or distinguish real Chinese characters from a set of pseudocharacters, non-characters, and visual symbols (Tong et al., 2009). Chinese OA tasks involve judging and memorizing the correct position of Chinese characters’ radical/structures. Tong and McBride-Chang (2010a) found visual-orthographic skills was a consistent factor in explaining both Chinese and English word reading of Hong Kong children.

Finally, RAN has been found to be another important determinant of reading ability across languages (McBride-Chang and Manis, 1996; Moll et al., 2009; Georgiou et al., 2013; Wang et al., 2014; Zhou and McBride, 2015). RAN is a microcosm of the processes of reading (Norton and Wolf, 2012), which measures a reader’s efficiency in accessing lexical elements that are frequently used in a language (e.g., characters, numbers, and colors). It indicates not just the quickness of naming but also the language’s oral proficiency (Zhou and McBride, 2015). Wolf and Bowers (1999) proposed PA and RAN double-deficit hypothesis for dyslexia in English based on a review of previous research. Shu et al.’s (2008) study showed that RAN was an effective predictor of Chinese character recognition.

In previous meta-analyses of Chinese as a first language, the relationship between PA, MA, RAN, and Chinese word reading have been estimated. Song et al. (2016) and Ruan et al. (2018) found moderate relationships between PA and Chinese word reading (0.36 and 0.302 with reading accuracy, respectively; 0.39 and 0.263 with reading fluency, respectively). Similarly, moderate relationships have been found between MA and Chinese word reading (0.393 with reading accuracy, 0.385 with reading fluency) (Ruan et al., 2018). In addition, the correlation between RAN and reading accuracy was −0.38, the correlation between RAN and reading fluency was −0.51 (Song et al., 2016).

### Cognitive Skills in L2 Chinese Character Reading

Regarding reading-related skills of Chinese as a second/foreign language, previous studies have controversial results. For the relationship between PA, MA, and Chinese L2 reading, some studies showed PA had moderate correlation with Chinese reading [e.g., 0.54 in Zhou et al. (2018)’s study; 0.57 in Hao and Zhang (2006)’s study; 0.41 in Ju et al. (2021)’s study; 0.3 in Zhang and Roberts (2021)’s study; 0.64 with character reading and 0.69 with two-character compound words reading in Hao and Wang (2020)’s study], while other studies showed no correlation [e.g., −0.06 in Zhou and McBride (2015)’s study; 0.33 in Chang (2011)’s study; 0.14 between tone awareness and character reading in Zhang (2017)’s study; 0.03 between onset-rime awareness and reading fluency, 0.29 between tone awareness and reading fluency in Hao and Zhou (2019)’s study].

It is worth noting that this inconsistency does not appear to be the result of the specific measures. This is because in the inconsistent results, some studies used similar types of measurements. For instance, Ju et al. (2021)’s study adapted from the PA measures used in Zhou and McBride (2015)’s study, the task included syllable deletion and phoneme deletion. However, these two studies showed different results. Zhou and McBride (2015)’s study had a non-significant correlation between PA and Chinese word reading (*r* = −0.06), while Ju et al. (2021)’s study presented a significant correlation (*r* = 0.41). In addition, the participants’ L1 was English in Ju et al. (2021)’s study, as was that of the majority of participants’ L1 in Zhou and McBride’s study (67.5% English). Such inconsistencies have also emerged in other studies [e.g., studies in Hao and Zhou (2019) and Hao and Wang (2020); studies in Hao and Zhang (2006) and Chang (2011)] (Hao and Zhang, 2006; Chang, 2011; Zhang, 2017; Zhou et al., 2018; Hao and Zhou, 2019; Hao and Wang, 2020; Ju et al., 2021; Zhang and Roberts, 2021).

In addition, the similar discrepancy was found in the correlation between MA and Chinese L2 reading, i.e., moderate correlation in some studies (Chang, 2011; Hao and Wang, 2020) and no significant correlation in others (Zhou et al., 2018). Zhang found that morphological compounding was not significantly correlated with both character reading (*r* = 0.172) and two-character compound words reading (*r* = 0.120), while the correlation between homography of MA and character/two-character word reading was significant (0.420 with character reading, 0.446 with two-character reading) (Zhang, 2017). Hao and Zhou’s (2019) study showed OA was correlated with reading accuracy and reading fluency while RAN just correlated with reading accuracy but not reading fluency.

As a result, it is important to investigate the controversy further based on larger samples and quantitative analyses in a meta-analysis with more existing research. In addition, our study can contribute to a better understanding of Chinese acquisition for CSL/CFL learners, which in turn helps researchers develop better CSL/CFL teaching methods and promote the development of Chinese teaching in the world.

### Age, Length of Study, First Language and Current Meta-Analysis

Age plays an essential role in language learning (Flege et al., 1995). Critical period hypothesis (Scovel, 1988; Abello-Contesse, 2009), multiple critical period hypothesis (Schouten, 2009; Deyeyser et al., 2010) and multiple sensitive period hypothesis (Long, 1990, 2005; Moyer, 2004) all illustrate the importance of age in second language acquisition. Moreover, for native Chinese speakers, prior studies have shown that different levels of PA might develop at different rates and explain Chinese reading skills differently with age (Li et al., 2012), and MA plays an increasing role in reading with age (Xue et al., 2013). Likewise, it has been demonstrated that in Chinese L2 reading, age has an impact on the Chinese acquisition (Chai, 2013). For example, in children’s studies, some showed significant correlations (e.g., PA and reading: Zhou et al., 2018’s study; MA and reading: Hao and Wang, 2020’s study) while others showed no correlations (e.g., PA and reading: Zhou and McBride, 2015’s study; MA and reading: Zhou et al., 2018’s study). These controversial results might be caused by age. Therefore, it is necessary to explore the moderating role of age. Along these lines, we tried to check the role of CSL/CFL learners’ age on Chinese reading.

Apart from age, length of residence is apparently another important predictor in L2 acquisition (Flege et al., 1999; Deyeyser et al., 2010). As many participants did not reside in China, it was more appropriate to use length of study as a moderator variable in our analysis. Additionally, we were interested in the role of different first languages on the relationship between cognitive skills and reading, so we also tried to test the moderating effect of the L1.

In summary, the present study aimed to explore cognitive skills for learning to read Chinese as a second language. Specifically, the purpose of this meta-analysis was to examine the strength of the relationship between Chinese PA, MA, OA, RAN and Chinese word reading in CSL/CFL learners. We aimed to answer the following questions:

(1) What is the magnitude of the association between Chinese PA, MA, OA, RAN and Chinese word reading for CSL/CFL learners?
(2) Do age, length of study and L1 play moderating roles in the relationship between cognitive skills and Chinese word reading?

## Method

### Eligibility Criteria

**FIGURE 1:**
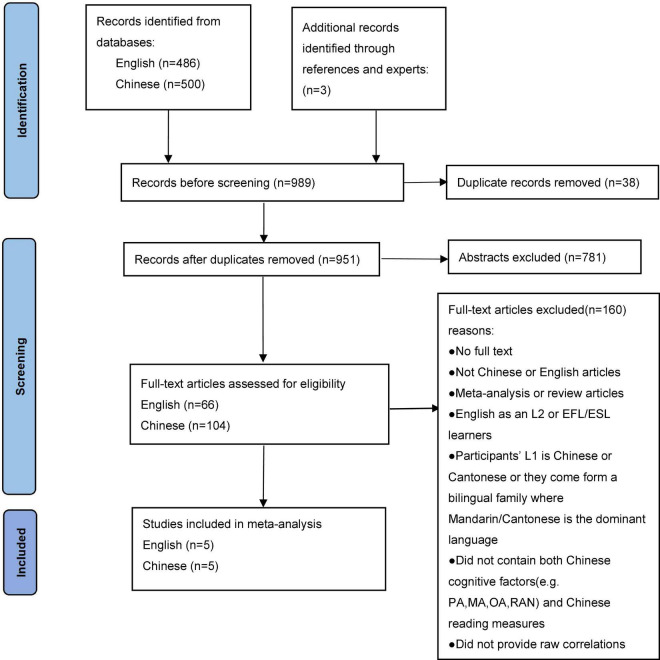
Flow diagram for the search and inclusion of studies (adapted from http://www.prisma-statement.org/).

The search and coding procedures are detailed in Figure 1. A series of inclusion and exclusion criteria were established to determine whether articles were included in this meta-analysis before the retrieval was formalized. The following criteria must be followed for the inclusion of studies: (a) studies assessed the correlations between at least one of PA, MA, OA, RAN, and Chinese word reading; (b) participants were CSL/CFL learners or bilinguals whose second language is Chinese; (c) to avoid any misunderstanding of the scripts, studies must be published in English or Chinese.

To avoid duplicate samples, articles by the same author were further screened. Only one study was coded for the data from the same group of participants. To be considered a measure of Chinese PA, the task should involve manipulation, generation, or judgment of syllables, phoneme, onset, rhymes, or tone in Chinese words (e.g., syllable deletion, phoneme deletion, onset-rime judgment, and the oddity test). In one of the studies, the participants were L2 Chinese-speaking ethnic minority children in Hong Kong, so the Cantonese phonological awareness and morphological awareness was tested as predictor variables (Zhou et al., 2018). Chinese MA included morphological construction, compounding production, homographic discrimination, and morphological judgment. In turn, to be considered a measure of Chinese OA, the test should involve judging and memorizing the correct position of Chinese characters’ radical/structures. Finally, RAN testing should require rapid array naming of objects, colors, characters, digits or other symbols in Chinese. The RAN task incorporated in our meta-analysis was for subjects to quickly read out Chinese characters or numbers, and then the time spent for oral reading was calculated. For Chinese word reading, the test included reading accuracy, reading fluency or overall reading skills. Test materials include single Chinese characters and two-character words.

### Data Collection Process

We first searched in computerized databases (Web of Science, Springer, Elsevier ScienceDirect, and ProQuest) for studies published in English using the subject words combined with related free words: Chinese as a second/foreign language OR CSL/CFL learners OR L2 Chinese AND cognitive factors OR phon* awareness OR phonological awareness OR PA OR morphological awareness OR MA OR orthographic awareness OR orthography OR OA OR rapid automatized* naming OR naming speed OR RAN. Then we searched in databases (CNKI, VIP, and WanFang data) for studies published in Chinese using Chinese translations of the aforementioned subject and free words: 汉语作为第二语言 OR CSL/CFL OR 汉语二语 OR 对外汉语 AND 认知 OR 语音意识 OR 语素意识 OR 正字法 OR 快速命名 OR 命名速度. There is no limit on the year of publication. Together with the three additional articles (checking the references following the articles and searching for articles by experts in the field), we retrieved a total of 989 articles. Ten articles, forty-two effect sizes, comprising 1103 subjects met the criteria for meta-analysis and were included in the final meta-analysis (see Supplementary Appendix).

### Coding Procedure

These studies were coded based on: (1) Basic study information: author(s), publication year, and publication type; (2) Sample sizes; (3) Age of participants; (4) Number of years participants had been learning Chinese (length of study); (5) Participants’ native languages (L1); (6) Measurement type of cognitive factors; (7) Reading type; (8) Effect sizes (*r* of PA, MA, OA, RAN, and reading). Detailed information regarding results coding is available in Supplementary Appendix.

### Meta-Analytic Procedures

The analyses followed standard analytic procedures as claimed in PRISMA^2^. The correlations between predictor variables (PA, MA, OA, and RAN) and Chinese word reading, as well as information pertinent to age and length of study were coded. We used the meta package in R software (version 4.0.3) to perform data analysis.

The effect sizes for the studies were displayed by the Pearson’s *r* correlation coefficient, and all correlation indicators were translated to Fisher’s *Z* scale, all analyses were run with the transformed data. For presentation, the data were transformed back to correlation coefficients, including the overall effect and its confidence interval. A 95% confidence interval (CI) was calculated for each effect size to examine whether the correlation was significantly different from zero. The overall correlation was estimated by calculating a weighted average of the correlations from each study, and the strength can be assessed by these general guidelines: 0.1 < | *r*| < 0.3, small/weak correlation; 0.3 < | *r*| < 0.5, medium/moderate correlation; | *r*| > 0.5, large/strong correlation (Cohen, 1988). The fixed-effect model (common-effect model) assumes that there is one true effect size and that all differences are caused by sampling error, and the random-effects model posits the effect size varies from study to study (Borenstein et al., 2009). Hence, our study used random-effects models for analysis. Then we identified and quantified heterogeneity through *Q* statistic (*p*) and *I*^2^ value (0–100%), if *I*^2^ is large, we should explain the reasons for the variance, for example, by using subgroup analysis to explain it. Higgins et al. (2003) suggested *I*^2^ values on the order of 25%, 50%, and 75% might be considered as low, moderate, and high. The leave-one-out method was used as a metric of sensitivity analysis to evaluate the stability and reliability.

For the categorical moderator variables (age and length of study), the studies were separated into subgroups based on the categories of the moderator variable. The degree of differences between the subgroups of studies was tested with a *Q* statistic and by comparing the correlation magnitude with CIs between the study subgroups. *Q*-test would be significant when between-groups difference is statistically larger than within-group difference.

A forest plot indicated the effect sizes and confidence intervals for each study. The horizontal line represented the confidence interval of the study results, the square showed the effect size of a single study, and the diamond represented the summary effect in our meta-analysis.

A funnel plot was used to test for publication bias, with the horizontal axis being the effect size, the vertical axis being the standard error and the vertical line in the middle being combined effect value, ideally the studies should be evenly distributed on both sides. In the presence of bias, the funnel will be asymmetric. The funnel plot is a subjective qualitative method to determine the presence or absence of bias, and therefore requires a statistical test for the degree of asymmetry in the funnel plot. We used Egger’s regression intercept test to address publication bias in studies (Egger et al., 1997), and whether the bias was statistically significant based on the *p*-value. If publication bias exists, the trim and fill method is used to correct the model (Duval and Tweedie, 2000a,b).

### Moderator Variables

We coded moderators: age, length of study and first language of the participants. The analysis for the moderator was not reported when there were less than four studies. As participants in some studies were from different countries and their L1 belonged to different language families, we had attempted a subgroup analysis using the language family to which their L1 belonged (Indo-European and Altaic). Only studies reported that participants from the same language family were included. Lamentedly, there were too few subgroup studies (less than four studies in the Altaic) to allow for L1 subgroup analysis. For age and length of study, it is noteworthy that the two moderator variables, did not overlap in our study, because participants in some studies were adults, but they had been learning Chinese for only a few months. In other studies, participants were children, and they had been learning Chinese since kindergarten (more than 3 or 5 years of learning). Considering this complex situation, that is why we conducted two different subgroup analyses. Ultimately, we coded two moderators (age and length of study) in the studies of the relationship between PA, MA, and Chinese character reading.

### Age

Since there is a strong correlation between age of acquisition and age at testing (Deyeyser et al., 2010), and some of the studies did not clearly report the age of acquisition of the participants, we used age at testing as a moderator variable. We divided two subgroups, children and adults. On one hand, as age 12 was often mentioned as a turning point in early literature (Lenneberg, 1967; McDonald, 2006; Abrahamsson and Hyltenstam, 2008), on the other hand, the age distribution of the participants in these studies showed a clear cut-off point at age 12–17 (no articles in the age range). Specifically, the age range of children subgroup was from 7 to 11 years old, and 18 to 26 years old of adults subgroup.

### Length of Study

There was also a cut-off point for the length of study of participants in these articles. Therefore, the L2 Chinese-speaking participants were divided into two subgroups according to the time they were exposed to the Chinese language environment. Beginning learners group learned Chinese for less than 2 years. Advanced learners group learned Chinese for 3–5 years.

## Results

The literature search and screening process resulted in 10 studies: five studies in Chinese and five studies in English. Forty-two effect sizes, comprising 1103 subjects met the criteria for meta-analysis and were included in the final meta-analysis, see Supplementary Appendix, for a list of the studies (e.g., age, length of study of the participants, measurement type, subgroup).

### Correlations Between Phonological Awareness and Word Reading of Chinese as a Second/Foreign Language

**FIGURE 2:**
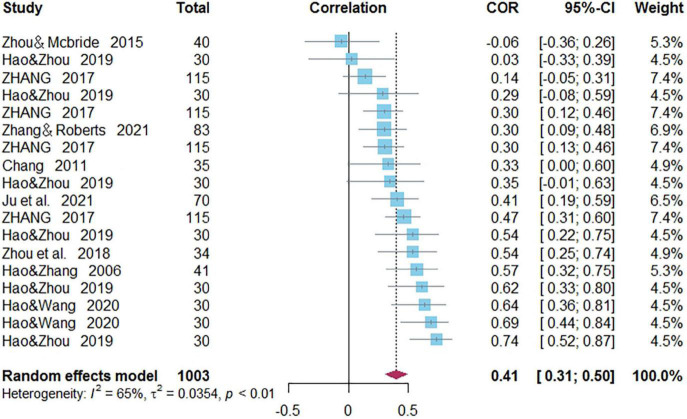
Forest plot of the correlations between phonological awareness and Chinese word reading.

**FIGURE 3:**
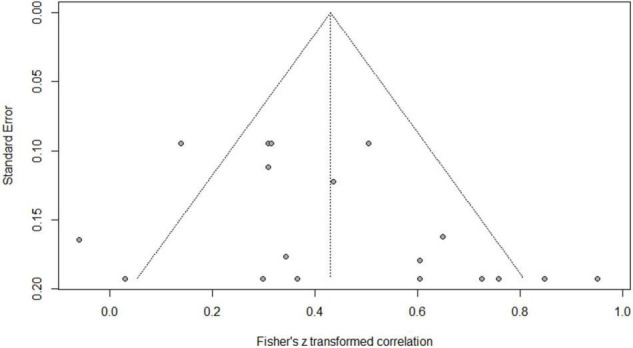
Funnel plot of effect sizes vs. standard error for phonological awareness and Chinese word reading.

Eighteen independent correlations including 1,003 subjects revealed the relationship between PA and Chinese word reading of CSL/CFL (Figure 2). The random-effects model used in this study, *Q*(17) = 47.92, *p* < 0.0001, *I*^2^ = 64.5% (Table 1). The weighted mean correlation was moderate and significant, *r* = 0.406, 95% CI [0.308,0.495], *Z*(17) = 7.50, *p* < 0.0001. The leave-one-out method was utilized to analyze reliability of the overall meta-analysis, revealing that each study made a similar contribution to the main effects, and the overall correlation raged from *r* = 0.38, 95% CI [0.29,0.49], to *r* = 0.43, 95% CI [0.33,0.51]. The funnel plot indicated no publication bias in the results (Figure 3). Egger’s regression intercept was 0.099, *t* = 1.83, *p* = 0.09, indicating that the correlation effect size did not have significant bias.

**TABLE 1:** Number of effect sizes, heterogeneity statistics, and Egger’s test of the relationship between phonological awareness, morphological awareness, orthographic awareness, rapid automatized naming, and Chinese word reading.

Predictor Variables	*k*	Effect size				Heterogeneity			Egger’s test			
		*r*	95% CI	*Z*	*p*	*Q*	*I* ^2^	*P*	Intercept	*SE*	*t*	*p*
PA	18	0.406	[0.308,0.495]	7.50	<0.0001	47.92	64.5%	<0.0001	0.099	0.168	1.83	0.09
MA	10	0.361	[0.218,0.489]	4.74	<0.0001	33.02	72.7%	0.0001	0.136	0.252	0.88	0.40
OA	9	0.376	[0.240,0.497]	5.14	<0.0001	24.79	67.7%	0.0017	0.312	0.243	0.32	0.76
RAN	5	−0.323	[−0.491, −0.133]	−3.25	0.0011	6.80	41.2%	0.15	0.020	1.607	−0.22	0.84

*CI, confidence interval; I^2^, the proportion of total variation between the effect size caused by real heterogeneity rather than chance.*

### Correlations Between Morphological Awareness and Word Reading of Chinese as a Second/Foreign Language

**FIGURE 4:**
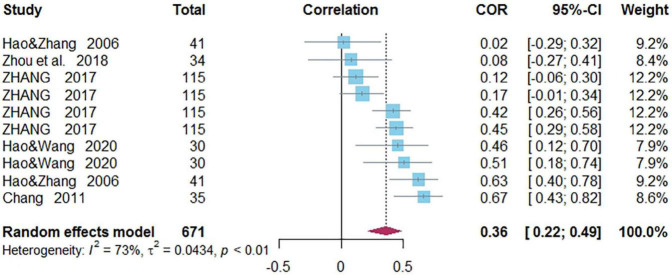
Forest plot of the correlations between morphological awareness and Chinese word reading.

**FIGURE 5:**
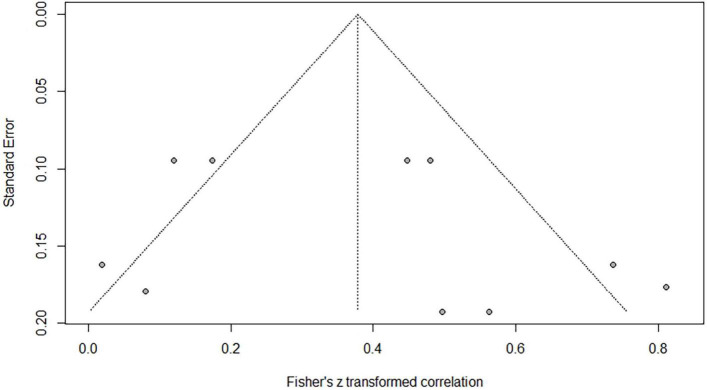
Funnel plot of effect sizes vs. standard error for morphological awareness and Chinese word reading.

Ten independent correlations including 671 subjects revealed the relationship between MA and Chinese word reading of CSL/CFL (Figure 4). The random-effects model used in this study, *Q*(9) = 33.02, *p* = 0.0001, *I*^2^ = 72.7% (Table 1). The weighted mean correlation was moderate and significant, *r* = 0.361, 95% CI [0.218,0.489], *Z*(9) = 4.74, *p* < 0.0001. The leave-one-out method was utilized to analyze reliability of the overall meta-analysis, revealing that each study made a similar contribution to the main effects, and the overall correlation raged from *r* = 0.32, 95% CI [0.18,0.45], to *r* = 0.39, 95% CI [0.23,0.52]. The funnel plot indicated no publication bias in the results (Figure 5). Egger’s regression intercept was 0.136, *t* = 0.88, *p* = 0.40, indicating that the correlation effect size did not have significant bias.

### Correlations Between Orthographic Awareness and Word Reading of Chinese as a Second/Foreign Language

**FIGURE 6:**
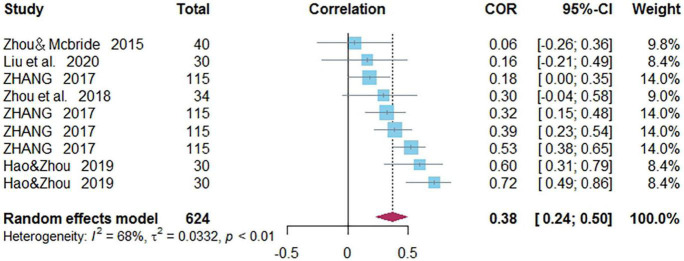
Forest plot of the correlations between orthographic awareness and Chinese word reading.

**FIGURE 7:**
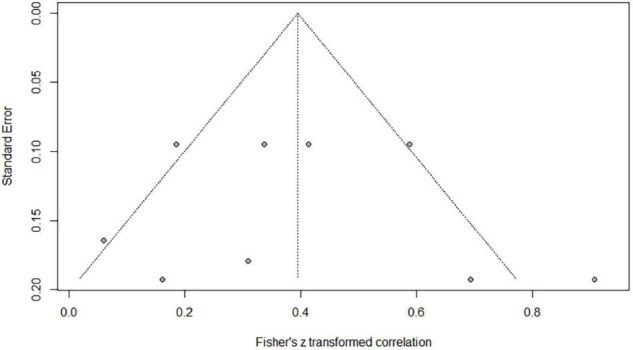
Funnel plot of effect sizes vs. standard error for orthographic awareness and Chinese word reading.

Nine independent correlations including 624 subjects revealed the relationship between OA and Chinese word reading of CSL/CFL (Figure 6). The random-effects model used in this study, *Q*(8) = 24.79, *p* = 0.0017, *I*^2^ = 67.7% (Table 1). The weighted mean correlation was moderate and significant, *r* = 0.376, 95% CI [0.240,0.497], *Z*(8) = 5.14, *p* < 0.0001. The leave-one-out method was utilized to analyze reliability of the overall meta-analysis, revealing that each study made a similar contribution to the main effects, and the overall correlation raged from *r* = 0.34, 95% CI [0.21,0.45], to *r* = 0.41, 95% CI [0.27,0.52]. The funnel plot indicated no publication bias in the results (Figure 7). Egger’s regression intercept was 0.312, *t* = 0.32, *p* = 0.76, indicating that the correlation effect size did not have significant bias.

### Correlations Between Rapid Automatized Naming and Word Reading of Chinese as a Second/Foreign Language

**FIGURE 8:**
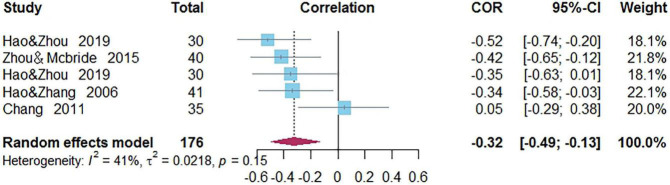
Forest plot of the correlations between rapid automatized naming and Chinese word reading.

**FIGURE 9:**
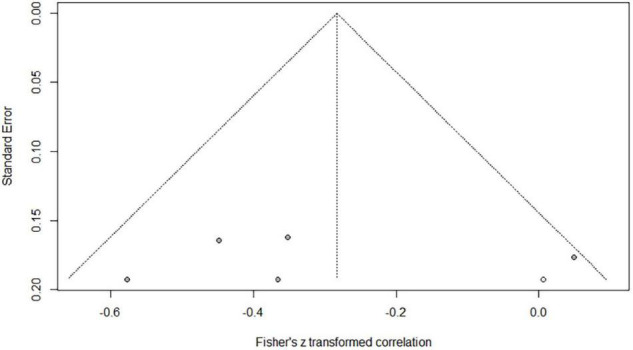
Adjusted funnel plot of effect sizes vs. standard error for rapid automatized naming and Chinese word reading.

Five independent correlations including 176 subjects revealed the relationship between RAN and Chinese word reading of CSL/CFL (Figure 8). The random-effects model used in this study, *Q*(4) = 6.80, *p* = 0.15, *I*^2^ = 41.2% (Table 1). The weighted mean correlation was moderate and significant, *r* = −0.323, 95% CI [−0.491, −0.133], *Z*(4) = −3.25, *p* = 0.001. The leave-one-out method was utilized to analyze reliability of the overall meta-analysis, revealing that each study made a similar contribution to the main effects, and the overall correlation raged from *r* = −0.40, 95% CI [−0.54, −0.25], to *r* = −0.28, 95% CI [−0.46, −0.07]. In addition, the funnel plot indicated that studies were missing on the right side of the mean. In the trim-and-fill analysis, one study was added (Figure 9) and the adjusted overall correlation was *r* = −0.276, 95% CI [−0.447, −0.085], *Z*(5) = −2.81, *p* = 0.005. Quantifying heterogeneity: *Q*(5) = 9.47, *p* = 0.092, *I*^2^ = 47.2%.

In addition, we compared the differences between these four correlation coefficients and found that they were not statistically significant (PA-reading vs. MA-reading: *Z* = 1.056, *p* = 0.291; PA-reading vs. OA-reading: *Z* = 0.693, *p* = 0.488; PA-reading vs. RAN-reading: *Z* = 1.164, *p* = 0.245; MA-reading vs. OA-reading: *Z* = 0.311, *p* = 0.756; MA-reading vs. RAN-reading: *Z* = 0.505, *p* = 0.614; OA-reading vs. RAN-reading: *Z* = 0.703, *p* = 0.482).

### Moderator Analyses

A subgroup analysis was used to test for moderating effects, the results of the moderator analyses were shown in Table 2.

**TABLE 2:** Test for moderating effects of age and length of study on Chinese word reading and phonological awareness/morphological awareness.

Moderator variable		*k*	*r*	95% CI	*Q*_*W*_	*I* ^2^	*Q*_*B*_	*p*
**Correlation between PA and CWR**	**Age subgroup**							
	Children	7	0.311	[0.181, 0.430]	16.26	63.1%	3.54	0.060
	Adults	11	0.483	[0.348, 0.598]	24.28	58.8%		
	**Length of study subgroup**							
	Beginning learners	12	0.487	[0.365, 0.593]	24.57	55.2%	5.20*	0.023
	Advanced learners	6	0.285	[0.150, 0.408]	13.65	63.4%		
**Correlation between MA and CWR**	**Age subgroup**							
	Children	5	0.270	[0.110, 0.416]	12.91	69.0%	2.07	0.150
	Adults	5	0.480	[0.228, 0.671]	14.08	71.6%		
	**Length of study subgroup**							
	Beginning learners	6	0.421	[0.172, 0.620]	18.98	73.7%	0.73	0.393
	Advanced learners	4	0.296	[0.124, 0.451]	11.44	73.8%		

PA, phonological awareness; MA, morphological awareness; CWR, Chinese word reading; CI, confidence interval; I^2^, the proportion of total variation between the effect size caused by real heterogeneity rather than chance; Q_W_, between-groups homogeneity of variance; Q_B_, within-group homogeneity of variance. *p < 0.05.

#### Age

**FIGURE 10:**
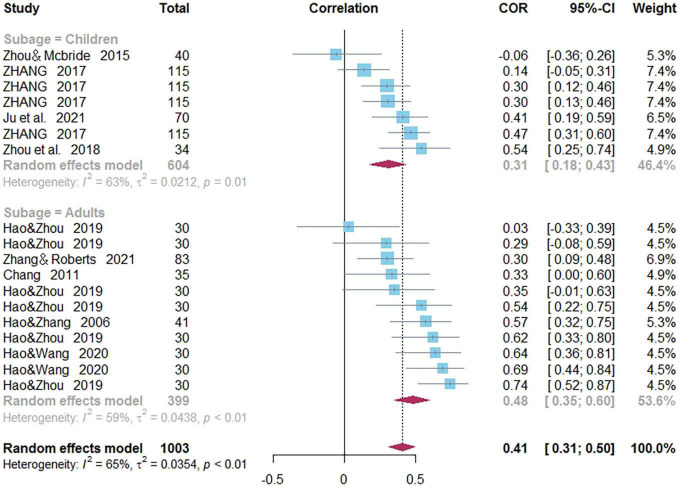
Forest plot for age subgroup analysis between phonological awareness and reading.

In the correlation between PA and reading, there are 7 effect sizes, comprising 604 subjects in children group, and 11 effect sizes, comprising 399 subjects in adults group. The moderating effect of age was marginally significant (*Q_*B*_* = 3.54, *p* = 0.060) (Figure 10): the correlation between PA and reading was slightly stronger in the CSL/CFL adults group (*r* = 0.483) than in the CSL/CFL children group (*r* = 0.311). In the correlation between MA and Chinese word reading, there are 5 effect sizes, comprising 494 subjects in children group, 5 effect sizes, comprising 177 subjects in adults group, and age did not play a moderating role (*Q_*B*_* = 2.07, *p* = 0.150).

#### Length of Study

**FIGURE 11:**
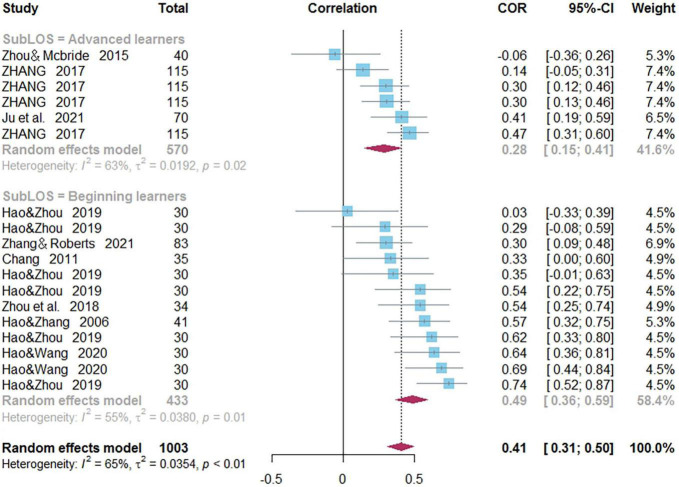
Forest plot for length of study subgroup analysis between phonological awareness and reading.

In the correlation between PA and reading, there are 12 effect sizes, comprising 433 subjects in beginning learners group, 6 effect sizes, comprising 570 subjects in adults group. A moderating effect of length of study on the correlation between PA and Chinese word reading was observed (*Q_*B*_* = 5.20, *p* = 0.023) (Figure 11): PA and reading correlated more strongly for beginning learners (*r* = 0.487) than for advanced learners (*r* = 0.285). In the correlation between MA and reading, there are 6 effect sizes, comprising 211 subjects in beginning learners group, 4 effect sizes, comprising 460 subjects in adults group, and length of study did not play a moderating role (*Q_*B*_* = 0.73, *p* = 0.393).

## Discussion

The present meta-analysis revealed moderate relations between reading-related skills (PA, MA, OA, and RAN) and Chinese word reading as a second/foreign language, and there were no significant differences among them. In addition, length of study moderates the relation between PA and reading.

First, our meta-analysis showed that PA correlated significantly with Chinese reading (*r* = 0.406), which supported the previous meta-analyses of Song et al. (2016) and Ruan et al. (2018) on the relationship between PA and reading in L1 Chinese. Although previous studies have shown that MA is more important than PA in Chinese reading (McBride-Chang et al., 2006, 2013; Shu et al., 2006), our study revealed that PA is equally crucial in Chinese reading, especially in Chinese as a second/foreign language.

In addition, our correlation coefficient is higher than Ruan et al. (2018) reported in their meta-analysis conducted in native Chinese subjects (reading accuracy: *Z* = 3.46, *p* < 0.001; reading fluency: *Z* = 0.97, *p* = 0.33). The difference might be due to the limited number of the present study and the different age groups between our study and Ruan et al.’s (2018) study. Ruan et al.’s (2018) meta-analysis excluded adults. If we only consider children subgroup, the *r* between PA and reading of the present study is similar to the size in their study (reading accuracy: *Z* = 0.23, *p* = 0.82; reading fluency: *Z* = 3.40, *p* < 0.001). In comparison with Wang et al.’s (2006a) study of L1 Chinese, L2 English bilinguals, our correlation coefficient for PA and reading is also larger (onset: *Z* = 2.43, *p* = 0.02; tone: *Z* = 2.58, *p* = 0.01). An explanation for this could be the cross-language transfer. Wang et al. (2006a,b, 2009) found that PA could be transferred from one language to another. That is to say cross-language facilitation in bilingual reading acquisition might occur at the phonological level. In our meta-analysis, most of the participants’ L1 were alphabetic languages. As previously stated, the relationship between PA and Chinese word reading could be facilitated by PA of the native language of CSL/CFL learners, especially for participants whose native language is an alphabetic language, as previous studies have shown a stronger correlation between PA and reading in alphabetic languages (McBride-Chang et al., 2006, 2013; Shu et al., 2006). Another explanation for this may be, most CSL/CFL learners start learning Chinese with Pinyin, which can facilitate the transfer of PA between Chinese and English reading (Wang et al., 2009). According to previous research, Chinese readers who had learned to read using Pinyin (e.g., Children in mainland China) were more adapted to manipulating phonics than readers who simply knew Chinese characters (e.g., Hong Kong children are not directly exposed to a phonetic coding system, they neither learn Pinyin like mainland children, nor do they learn Zhuyin Fuhao like Taiwanese children) (Read et al., 1986; McBride-Chang et al., 2004). For CSL/CFL learners, the study of Pinyin promoted cross-language transfer.

It should be noted that we also observed that length of study moderated the relationship between PA and CSL/CFL reading, and age’s effect was marginally significant. The correlation observed in beginning learners was stronger than advanced learners. Among native Chinese readers, Song et al. (2016) found age/grade did not moderate the relationship between PA and Chinese word reading, but the participants in their study were all children, with a grade range of 1–6. In contrast, the age distribution of the participants in our study was much wider. According to the multiple critical period hypothesis (Deyeyser et al., 2010), for adult learners, there is a significant association between ultimate attainment and verbal aptitude, but not for kids. Liu et al. (2020) found PA contributed significantly to Chinese character reading of beginning learners (studied Chinese for about 6 months) than zero-starting learners (studied Chinese for less than 3 months). Our study extends the age range further. Non-native speakers learn Chinese mostly through pinyin at the beginning. As we discussed before, beginning learners are influenced by the cross-language transfer effect, which can facilitate the link between PA and reading (Wang et al., 2009). However, as the length of study increases, L2 Chinese learners become more proficient in Chinese reading. Thus, for advanced learners, their Chinese reading has even reached an automated level, which makes them less dependent on PA. This may be the reason why their PA and reading correlation are lower than those of the beginning learners.

Secondly, our results also showed MA was significantly related to Chinese word reading (*r* = 0.361), which was similar to the previous meta-analysis of Ruan et al. (2018) (0.385 with reading fluency and 0.393 with reading accuracy). It is worth mentioning that according to previous studies, PA was more important for reading in English than for reading in Chinese, while MA was more important for reading in Chinese than English (McBride-Chang et al., 2005, 2006; Shu et al., 2006). Our results showed there was no significant difference in *r* between PA-reading and MA-reading (*Z* = 1.056, *p* = 0.291). It indicates that MA is equally important as PA in reading Chinese as a second language. As we have previously described, for CSL/CFL learners, especially those whose native language are alphabetic languages, the transfer of their GPC rules is more likely to facilitate the relationship between PA and Chinese reading than between MA and Chinese reading. In addition, age and length of study did not moderate the relationship between MA and reading. This is consistent with the finding of previous studies (McBride-Chang et al., 2003; Ruan et al., 2018). Ruan et al. (2018) coded grade level instead of age to distinguish reading development phases. They coded kindergarten children as “preschooler,” Grade 1 and 2 as “beginning,” Grade3 and 4 as “intermediate,” Grade 5 and above as “advanced.” They found grade did not moderate the correlation between MA and Chinese reading. These findings suggest that the correlation between MA and Chinese word reading was stable across age and learning stages, and MA is important for Chinese reading among CSL/CFL learners.

Thirdly, the results of our meta-analysis also revealed a significant relationship between OA and Chinese word reading (*r* = −0.376). Tong and McBride-Chang (2010a) investigated the relationships of OA, PA, and MA to Chinese and English word reading in Hong Kong children learning English as a second language. They discovered that OA was modestly related to Chinese word reading (*r* = 0.29 in 5th grade and *r* = 0.41 in 2nd grade). In addition, they found that Chinese OA could predict English reading (Tong and McBride-Chang, 2010a). We can infer that for L2 Chinese learners, their native OA may also facilitate the connection between Chinese OA and reading.

Finally, our findings showed that RAN was significantly related to Chinese word reading (*r* = −0.323). In the previous meta-analysis of Chinese and English (Araújo et al., 2015; Song et al., 2016), RAN correlated significantly with both reading accuracy (Chinese: *r* = −0.38, English: *r* = 0.42) and reading fluency (Chinese: *r* = −0.51, English: *r* = 0.49). RAN is a microcosm of the processes of reading, so it is likely to be related to reading fluency rather than accuracy (Norton and Wolf, 2012). Our correlation coefficient among CSL/CFL is smaller than those of previous studies in native Chinese readers, which may be due to the specificity of the subject population. A cross-language study found that L2 Chinese learners had a slower speed of Chinese RAN compared with native Chinese speakers (Zhou and McBride, 2015). For Chinese, syllable-character mapping is more arbitrary (Shu et al., 2003; Zhou and McBride, 2015). If learners do not know the meaning of a Chinese character, there are literally very few clues to rely on. The RAN task incorporated in our meta-analysis was for subjects to quickly read out Chinese characters or numbers, and then the time spent for oral reading was calculated. Obviously, CSL/CFL learners take more time than native speakers from seeing the target to speaking out. Furthermore, the correlation between RAN and Chinese reading in our meta-analysis is based on a smaller number of studies. As a result, our findings should be interpreted with caution and follow-up studies can continue to discuss the relationship between RAN and reading ability in second language learners.

### Limitations

First, previous studies have concluded that learning to read Chinese relies on PA abilities far less than learning to read English does (Huang and Hanley, 1995; Ruan et al., 2018). However, in our study, we observed the importance of PA in Chinese reading as a foreign/second language. This may be due to the specificity of the participants. Additionally, in present study, most of the subjects’ L1 belonged to Indo-European, and only one article’s participants were Korean whose native language belonged to Altaic, so that a subgroup analysis of L1 could not be carried out. Similarly, subgroup analyses for Mandarin vs. Cantonese and character vs. word reading could not be performed due to limitations in the number of studies. Future studies could use these as moderating variables to examine whether transfer effects occur across different native languages if possible. Second, because of the relatively small number of extant articles, our age variable could not be subdivided among children, so we cannot make good inferences about whether cognitive factors play different roles at different stages for children CSL/CFL learners, which makes it difficult to compare with previous meta-analyses in Chinese children. Similarly, due to the limitation in numbers of articles, we were unable to divide the two aspects of reading (accuracy and fluency) as Song et al. (2016) and Ruan et al. (2018) did. Finally, the analysis for the moderator was not reported in OA and RAN, because there were not enough studies. Future studies can further investigate this issue when conditions allow.

### Educational Implications

Learning Chinese as a second/foreign language is becoming increasingly important worldwide, and research on CSL/CFL reading can promote the development of L2 Chinese teaching. Our findings can promote a better understanding of CSL/CFL learning and help to propose targeted language training based on the role of different cognitive skills on reading, thus improving learning efficiency and teaching effectiveness. Results also shed light on the impact of length of study on the influence from PA to reading, which provides implications for the sensitive period of phonological learning for CSL/CFL learners.

## Conclusion

Our meta-analysis explored the relationship between cognitive skills and reading form an L2 Chinese perspective, and showed that PA, MA, OA and RAN are significant correlates of Chinese reading. In addition, length of study moderated the relationship between PA and Chinese reading.

## Data Availability Statement

The original contributions presented in the study are included in the article/Supplementary Material, further inquiries can be directed to the corresponding author.

## Author Contributions

JZ: conceptualization, supervision, reviewing, and editing. XC: data analysis and writing-original draft preparation. Both authors contributed to the article and approved the submitted version.

## Conflict of Interest

The authors declare that the research was conducted in the absence of any commercial or financial relationships that could be construed as a potential conflict of interest.

## Publisher’s Note

All claims expressed in this article are solely those of the authors and do not necessarily represent those of their affiliated organizations, or those of the publisher, the editors and the reviewers. Any product that may be evaluated in this article, or claim that may be made by its manufacturer, is not guaranteed or endorsed by the publisher.

## Footnotes

1
http://www.gov.cn/xinwen/2020-09/05/content_5540872.html

2
http://www.prisma-statement.org/
